# AI-Driven Weather Data Superresolution via Data Fusion for Precision Agriculture

**DOI:** 10.3390/s26041297

**Published:** 2026-02-17

**Authors:** Jiří Pihrt, Petr Šimánek, Miroslav Čepek, Karel Charvát, Alexander Kovalenko, Šárka Horáková, Michal Kepka

**Affiliations:** 1 Faculty of Information Technology CTU in Prague, Thákurova 9, Praha 6, 160 00 Prague, Czech Republic; pihrtjir@fit.cvut.cz (J.P.); simanpe2@fit.cvut.cz (P.Š.); miroslav.cepek@fit.cvut.cz (M.Č.); alexander.kovalenko@fit.cvut.cz (A.K.); 2 Help Service–Remote Sensing s.r.o., Husova 2117, 256 01 Benešov, Czech Republic; 3 Lesprojekt-služby s.r.o., Martinov 197, 277 13 Záryby, Czech Republic; horakova@lesprojekt.cz; 4 WirelessInfo, z.s., Cholinská 1048/19, 784 01 Litovel, Czech Republic; kepka@wirelessinfo.cz

**Keywords:** precision agriculture, weather downscaling, superresolution, data fusion, near-surface air temperature, Global Forecast System (GFS), ERA5-Land, sensor networks, TabPFN, K-nearest neighbors (KNN)

## Abstract

**Highlights:**

**What are the main findings?**

- Multi-source data fusion (GFS predictors + station observations + static physiography) consistently improves 24 h 2 m air temperature forecasts relative to raw GFS across all spatiotemporal splits.
- The best operational configuration is TabPFN-KNN, achieving MAE = 1.26 °C in the most demanding regime (time = validation, space = validation), i.e., ≈24% lower error than GFS (1.66 °C).

**What are the implications of the main findings?**

- High-resolution, spatially continuous near-surface temperature fields can be generated from routinely available forecast inputs and regional station networks, supporting field-scale agricultural decisions.
- The hybrid design (station-level learning + physiography-conditioned KNN propagation) provides a deployable pathway for superresolution services integrated into sensor infrastructures (e.g., SensLog/ALIANCE).

**Abstract:**

Accurate field-scale meteorological information is required for precision agriculture, but operational numerical weather prediction products remain spatially coarse and cannot resolve local microclimate variability. This study proposes a data fusion superresolution workflow that combines global GFS predictors (0.25°), regional station observations from Southern Moravia (Czech Republic), and static physiographic descriptors (elevation and terrain gradients) to predict the 2 m air temperature 24 h ahead and to generate spatially continuous high-resolution temperature fields. Several model families (LightGBM, TabPFN, Transformer, and Bayesian neural fields) are evaluated under spatiotemporal splits designed to test generalization to unseen time periods and unseen stations; spatial mapping is implemented via a KNN interpolation layer in the physiographic feature space. All learned configurations reduce the mean absolute error relative to raw GFS across splits. In the most operationally relevant regime (unseen stations and unseen future period), TabPFN-KNN achieves the lowest MAE (1.26 °C), corresponding to an ≈24% reduction versus GFS (1.66 °C). The results support the feasibility of an operational, sensor-infrastructure-compatible pipeline for high-resolution temperature superresolution in agricultural landscapes.

## 1. Introduction

Global food systems face increasing pressure due to climate change and population growth. The world population is projected to reach 9.7 billion by 2050 [1], while climate change is associated with increased variability and a higher frequency of extreme weather events [2]. These trends increase the demand for accurate, spatially detailed meteorological information that can support risk-aware planning in agriculture. Precision agriculture frameworks underline that site-specific weather and microclimate information are a prerequisite for optimizing inputs, reducing environmental impacts, and stabilizing yields [3,4,5].

Operational numerical weather prediction (NWP) models such as the Global Forecast System (GFS; National Centers for Environmental Prediction, National Oceanic and Atmospheric Administration—NOAA, College Park, MD, USA) provide global coverage and multi-variable forecasts at regular update cycles. However, they operate at horizontal resolutions on the order of tens of kilometers; for example, standard GFS configurations use grid spacing of approximately 25–28 km [6]. At this scale, important microclimatic variations driven by the local topography, land cover, and surface heterogeneity are not resolved. Within a single NWP grid cell, the precipitation, temperature, and humidity can differ substantially between slopes, valleys, and plateaus [7]. Consequently, raw NWP products are often too coarse to directly support field-scale decision-making in agriculture.

Local meteorological stations and dedicated agricultural weather networks partially compensate for this limitation. Standardized in situ measurements provide accurate point data for key variables such as the air temperature, humidity, wind, and precipitation [8]. Agricultural meteorological networks and farm-scale monitoring systems extend this capability to specific crops and production systems [9]. Nevertheless, the spatial density of such stations is generally limited by installation and maintenance costs, and the available networks frequently provide monitoring rather than dedicated forecasting products. As a result, there is a gap between coarse-grid forecasts and the high-resolution weather information required at the field scale.

Hybrid data-driven approaches aim to bridge this gap by combining global model outputs, reanalysis products, and station observations. Quality-controlled station archives such as HadISD provide sub-daily time series from thousands of stations worldwide, including long-term records from Central Europe [10,11]. Global reanalysis products such as ERA5 and its land-focused derivative ERA5-Land offer physically consistent gridded data at a higher spatial resolution than operational NWP, produced by assimilating in situ and remote sensing observations into numerical models [12,13]. The use of multi-source observations and data assimilation has led to significant improvements in large-scale land–atmosphere representation [14]. For local forecasting and downscaling, these datasets can be used either as direct predictors, as high-resolution targets for supervised learning, or as auxiliary sources that encode information about the conditions in the surroundings of a station.

In previous work, we investigated the combination of local weather station data with global forecasts and reanalysis to improve 24 h-ahead predictions at station locations in the Czech Republic [15]. HadISD station data from 27 sites were combined with GFS forecasts on a 0.25° grid, and ERA5-Land reanalysis was used as an additional source of spatial context. Multi-layer perceptrons, CatBoost gradient boosting models, and long short-term memory (LSTM) neural networks were evaluated, and a U-Net-based model was trained to approximate ERA5-Land fields from GFS inputs, providing a high-resolution proxy in near-real time. The results showed that integrating station measurements with GFS outputs significantly reduced forecast errors compared to GFS alone, confirming the potential of hybrid local–global methods for agricultural applications.

From a methodological perspective, recent ML work relevant to this study can be organized into three complementary categories: (i) deep neural networks for spatial superresolution and downscaling, typically implemented as convolutional encoder–decoder models, learn non-linear mappings from coarse-grid NWP (or reanalysis) predictors to fine-grid fields and are widely used for meteorological reconstruction tasks [16,17,18]; (ii) gradient-boosted tree ensembles (e.g., CatBoost, LightGBM) provide strong tabular post-processing for station-level bias correction when heterogeneous predictors (NWP variables, recent observations, and site descriptors) are available [19,20]; and (iii) spatiotemporal architectures (e.g., CNN-LSTM hybrids, temporal convolutional networks, and Transformers) exploit the temporal context and can improve short-term forecasting and the temporally consistent post-processing of NWP outputs [21,22,23,24]. Complementary statistical tools such as copulas address multivariate dependence in hydrometeorological extremes [25], and convolutional networks have been used for extreme event detection in gridded reanalyses [26]. At the scale of climate and NWP ensembles, ML post-processing can capture situation-dependent biases and improve skill [23,27], with recent surveys summarizing applications across data assimilation, uncertainty quantification, and post-processing workflows and highlighting evaluation challenges for complex non-linear methods [21,24].

Recent advances in machine learning provide a suitable methodological framework for such hybrid systems. Deep learning has become a standard tool for modeling high-dimensional, non-linear relationships in large datasets [16]. In meteorology, hybrid deep models have been used for the short-term forecasting of temperature and precipitation [17,18]. Gradient boosting methods such as CatBoost and LightGBM have demonstrated strong performance in tabular regression tasks relevant to weather prediction, especially with heterogeneous predictor sets [19,20]. Dedicated spatiotemporal architectures, including convolutional neural network–LSTM hybrids and temporal convolutional networks, have been proposed for station-based time series and local weather variables [28,29]. Statistical approaches using copulas address multivariate dependence structures in hydrometeorological extremes [25], while convolutional networks have been successfully applied to detect extreme events in gridded climate reanalyses [26]. At the scale of climate and NWP ensembles, machine learning has been shown to improve predictive skill by post-processing and refining coarse model outputs [27].

Recent work specifically on the ML-based post-processing of NWP further reinforces its relevance: a recent technical survey summarizes applications of ML across data assimilation, uncertainty quantification, and post-processing workflows [21]. Transformer-based post-processing has also been demonstrated for quantitative precipitation estimation, yielding substantial skill improvements across rain intensity levels [22]. In addition, ML-based error models have been shown to capture situation-dependent biases and reduce the RMSE by about 10–15% across lead times and variables [23]. Recent discussions also emphasize the challenges of objectively measuring forecast skill for complex non-linear post-processing methods [24].

Beyond point predictions, considerable effort has been devoted to exploiting machine learning for the spatial downscaling and superresolution of Earth system data. Deep learning has been used both to emulate sub-grid-scale processes in climate models and to reconstruct fine-scale spatial patterns from coarse inputs [30,31]. Single-image superresolution approaches such as DeepSD have been applied to climate projections, demonstrating that convolutional encoder–decoder architectures can recover high-resolution fields from low-resolution climate information [32]. Deep learning-based gridded downscaling methods for surface meteorological variables in complex terrain further confirm that such architectures can handle strong orographic effects [33]. Enhanced residual U-Net designs have been proposed for the temporal and spatial downscaling of gridded geophysical data [34], building on the original U-Net architecture developed for image segmentation [35]. More recently, superresolution methods have been applied directly to global weather forecasts [36], showing that operational NWP outputs can be post-processed into higher-resolution fields with the improved representation of local structures.

These developments are closely connected to precision agriculture, where wireless sensor networks, Internet of Things infrastructures, and decision support systems are used to link environmental observations to crop and farm management [3,5,37,38,39]. Weather- and climate-informed decision-making is central to many precision agriculture applications, including frost protection, irrigation scheduling, disease risk modeling, and harvest logistics. Several recent studies explicitly combine machine learning-based forecasting with agricultural advisory use cases—for example, in digital agriculture platforms and regional decision support systems [38,40]. In practice, short-horizon (hours to one day) forecasts of the nocturnal minimum 2 m temperature and its timing are a primary input for frost protection because they indicate whether and when to deploy active mitigation (e.g., sprinklers, wind machines, heaters) and how to allocate labor and resources [41,42]. Spatially continuous, sub-kilometer temperature fields are particularly valuable in complex terrain, where cold-air pooling creates strong within-field gradients that are not resolved by coarse-grid NWP [41,42,43]. While robust advisory services also integrate the humidity/dew point, wind, and precipitation, temperature remains the first-order trigger for frost risk, and our workflow can be extended to additional near-surface variables as observations permit [41,42]. However, many existing systems remain limited either to station-level forecasts or to coarse-grid NWP products, without providing spatially continuous, high-resolution fields at scales directly relevant to fields and plots [41,43,44].

The operational deployment of such methods requires not only robust predictive models but also sensor data infrastructure that can ingest, harmonize, and disseminate data from multiple networks. The SensLog platform (International consortium (Czech Republic, Latvia)) was proposed as a general solution for managing heterogeneous sensor data, including environmental and citizen observatory measurements [37,38]. SensLog provides functionality for registering sensors, handling different communication protocols, storing time series, and exposing them through standardized interfaces. It has been applied in several environmental and agricultural contexts and is suitable as the data management backbone for machine learning-based forecasting services that need to handle distributed station networks.

Building on our previous station-level hybrid post-processing framework [15], which corrected NWP forecasts at sensor locations, the present work adds an explicit spatial superresolution layer that converts station-level corrections into spatially continuous, high-resolution temperature fields for precision agriculture. Compared with [15], the key extensions are (i) the introduction of a physiography-conditioned KNN propagation step to generate gridded products; (ii) an expanded benchmark including modern tabular and sequence models (LightGBM, TabPFN, Transformer, and BayesNF) and their KNN hybrid variants; and (iii) validation regimes that test generalization in both space and time. The model set was selected to represent complementary methodological families while remaining computationally feasible for operational deployment; the rationale for the selection is provided in Section 2.4.2. We focus on the near-surface air temperature, which is a primary driver for many agricultural processes and a key variable for frost risk. Building on publicly available GFS forecasts, ERA5-Land reanalysis, high-resolution topographic data, and dense agricultural weather station networks, we construct a data fusion pipeline that produces sub-kilometer temperature fields. Machine learning models are trained to map from coarse NWP predictors, reanalysis-based proxies, static geographical descriptors, and station observations to high-resolution temperature estimates, following recent advances in gradient boosting and deep neural architectures for geoscientific data [19,20,29,30,31,32,33,34,35,36]. The resulting framework is designed to be integrated with SensLog [37,38] and other operational services, enabling the practical deployment of superresolved weather information into agricultural decision support chains.

### Research Goals and Hypotheses

This study tests whether superresolution and multi-source data fusion can deliver operationally useful, field-scale near-surface air temperature information for precision agriculture when driven by routinely available forecast and sensor inputs. The research goals are to develop an operational pipeline that produces spatially continuous high-resolution temperature fields; to quantify the contributions of station observations, reanalysis proxies, and static physiographic descriptors relative to a global NWP baseline; and to implement an end-to-end workflow in which observations are ingested, quality-controlled, and served through a sensor platform such as SensLog for training and near-real-time inference.

The work evaluates three hypotheses. Hypothesis H1 states that models conditioned on global NWP predictors reduce the near-surface air temperature error relative to raw NWP at independent station locations. Hypothesis H2 posits that static physiography reduces location-dependent biases, particularly when generalizing to unseen locations, which is assessed via backbones with and without physiography-conditioned KNN propagation. Hypothesis H3 posits that the workflow maintains stable skill over seasons and under realistic station availability constraints. In this manuscript, H1, H2, and H3 are explicitly evaluated in Section 4.5.

These goals and hypotheses establish a testable structure for the remainder of the manuscript, covering baseline definitions and datasets in the Section 2, superresolution model formulation and ablation studies in the Methods and Results, statistical verification and robustness checks in the Results, and integration aspects with the sensor platform in the System and Implementation sections.

## 2. Materials and Methods

### 2.1. Study Area

**Figure 1:**
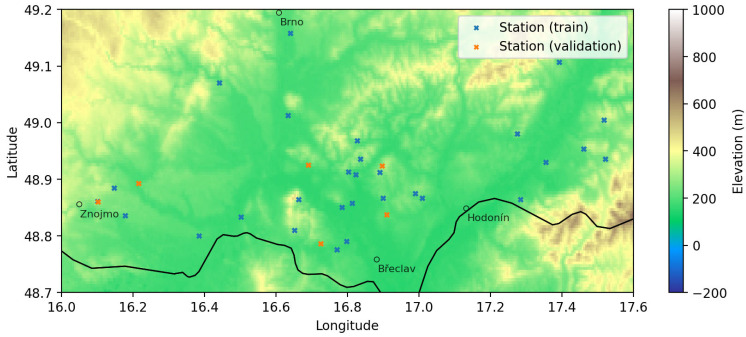
Study area—Southern Moravia region in the Czech Republic, covering approximately 60 × 120 km. Elevation, major cities, and the country border in the area are displayed for context.

The experiments were conducted in the wine-growing region of Southern Moravia (Czech Republic). This area is characterized by gently undulating terrain with elevations typically between 150 and 400 m a.s.l., heterogeneous land use dominated by vineyards and arable land, and pronounced microclimatic variability driven by the local topography (valleys, slopes, plateaus). To ensure sufficient spatial sampling of these microclimatic gradients, we used a dense network of automated agricultural weather stations operated by the Association of Integrated and Organic Production of Grapes and Wine (EKOVÍN, Czech Republic). The final dataset includes 36 stations selected based on temporal completeness, sensor calibration quality, and spatial representativeness within the region (Figure 1).

The stations record the near-surface air temperature at 15-min intervals, which allows us to resolve diurnal cycles and short-lived events such as nocturnal inversions and advective cold-air drainage. Additional variables (relative humidity, precipitation, leaf wetness, solar radiation) are available at many stations, but, for the present work, we use them only as auxiliary quality control information; the target variable for superresolved prediction is the 2 m air temperature.

### 2.2. Data

#### 2.2.1. Numerical Weather Prediction Data (GFS)

As the primary large-scale predictor, we use outputs from the Global Forecast System (GFS), a global numerical weather prediction model with horizontal grid spacing of 0.25° (≈28 km in mid-latitudes) and 6-hourly forecast cycles. From the NCAR Research Data Archive (National Center for Atmospheric Research, Boulder, CO, USA), we extract a set of surface and low-level variables including the 2 m air temperature, relative humidity, surface pressure, wind components, precipitation, and shortwave radiation. We select only 24-h lead time predictions (00 UTC + 24 h, 06 UTC + 24 h, 12 UTC + 24 h, 18 UTC + 24 h) for simplicity, but other lead times can be used as well.

These GFS fields serve two roles: (i) they define the baseline reference against which superresolution skill is evaluated, and (ii) they form a core subset of predictors for the machine learning models. For each station and forecast time, we sample the GFS grid cell containing the station and construct feature vectors that combine current and lagged GFS predictors with other inputs described below.

#### 2.2.2. Agricultural Weather Station Network

The vineyard station network provides high-frequency in situ observations, used both as training targets and, in some configurations, as autoregressive predictors. Each station is equipped with calibrated sensors for the near-surface temperature and other meteorological variables. Raw observations are first passed through the SensLog platform, which provides basic quality control (range checks, detection of missing or obviously erroneous values, e.g., implausible temperature extremes or abrupt discontinuities inconsistent with local meteorological conditions), timestamping, and continuous storage in a central database.

For the superresolution experiments, station data are aggregated to an hourly resolution to align with the temporal resolution of the GFS predictors. For each station, we retain only periods with continuous records over the training and validation intervals; stations with substantial gaps or sensor problems are excluded to avoid introducing spurious noise into the learning process. We select 30 stations for training and 6 stations for validation.

#### 2.2.3. Static Geographic Data

Static physiographic predictors provide spatial context that is essential for representing topographically induced temperature patterns at sub-kilometer scales. For each station and each target grid cell in the high-resolution prediction lattice, we derive the latitude and longitude, the elevation from the ASTER Global Digital Elevation Model (NASA, Washington, DC, USA; METI, Tokyo, Japan) at a 500 m resolution, and the north–south and east–west elevation gradients computed by finite differences to represent the slope magnitude and aspect-related effects.

These static features enable the models to learn systematic temperature differences between, for example, valley bottoms and surrounding slopes or between north- and south-facing slopes and are also used as inputs in the K-nearest neighbors (KNN) interpolation stage (Section 2.4.4).

Although the elevation data are available at a higher resolution, our experiments using the full-resolution data showed slightly lower performance. This could be attributed to noise in elevation or to slightly inaccurate coordinates for the weather stations in our dataset.

**Figure 2:**
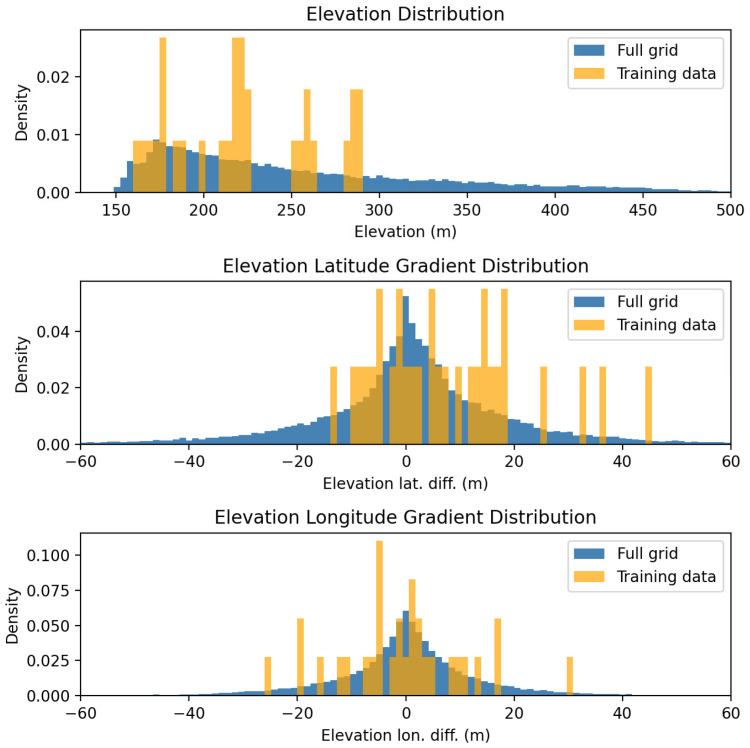
Static feature distributions for training data and full-resolution inference grid.

The specific elevation and slope values are calculated via linear interpolation at the station coordinates. The distribution of the training data, as well as the full-resolution inference grid, is shown in Figure 2.

#### 2.2.4. Data Integration and Feature Construction

All dynamic and static sources are integrated into a common tabular representation. For each station and time pair used in training or validation, we construct a feature vector that combines station-based predictors such as the recent temperature history and, where available, other meteorological variables aggregated over the preceding hours and GFS-based predictors such as the full set of selected surface and low-level fields at the station grid cell. We optionally include derived features such as helicity, the minimum temperature aboveground, and precipitable water, as well as static physiographic descriptors such as elevation and slope components, as described above.

To reduce the dimensionality, we adopt a compact predictor set guided by the feature importance/SHAP analysis reported in our earlier GFS–station fusion study [15], prioritizing variables known to be relevant for the near-surface temperature (e.g., surface temperature, precipitable water, station temperature, and dew point). For clarity, we do not repeat the SHAP analysis in this manuscript and therefore do not report SHAP plots here.

### 2.3. Overall System Architecture

#### 2.3.1. Logical Workflow

**Figure 3:**
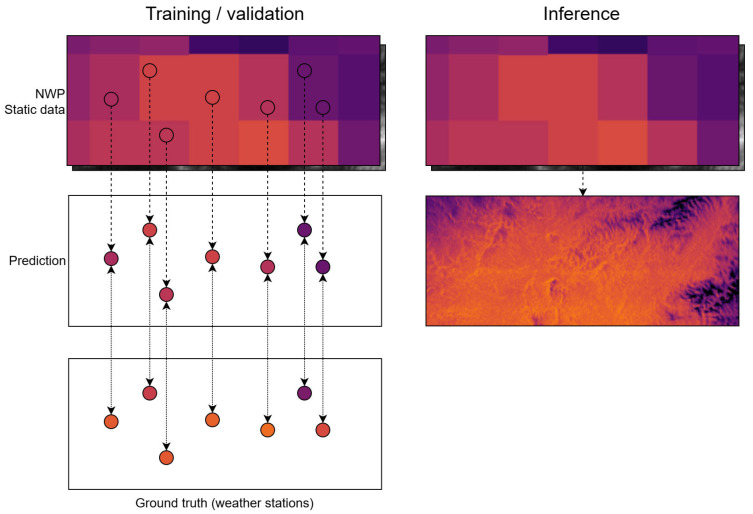
Method illustration: models are trained/validated only using values at weather station locations, but, during inference, the full spatial inputs can be used to produce predictions for any location. The figure illustrates this for a singular time step. Dots represent weather station locations; dotted arrows indicate model predictions, and solid arrows indicate comparison with the ground truth observations. The color scale illustrates temperature patterns; the exact numerical values are not relevant for this schematic illustration.

**Figure 4:**
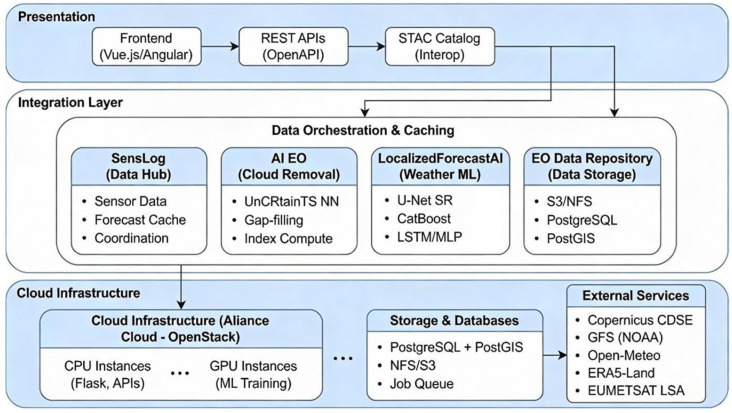
Overall ALIANCE infrastructure and dataflow of the end-to-end workflow.

The system architecture adopts the modular ALIANCE [37,38,45,46] platform approach. A schematic illustration of the training and inference concept is shown in Figure 3, while the overall architecture is summarized in Figure 4. The end-to-end workflow implemented in this study is represented in Figure 4 through the dataflow between components and is described here as five stages that cover acquisition and ingestion, pre-processing and feature assembly, model training, operational inference and superresolution, and publication and integration. External forecast data, such as GFS data, are downloaded on a rolling basis and stored, while station measurements are ingested into SensLog through feeder services that normalize formats and apply basic quality control. A pre-processing layer maps station locations to the GFS grid, merges dynamic predictors with static physiographic attributes, constructs feature vectors and targets, and prepares a high-resolution prediction grid by sampling static predictors at the desired resolution. Models are trained using historical data and evaluated under identical spatiotemporal cross-validation schemes. For a given forecast time, a trained model ingests the latest available GFS fields and relevant static attributes to produce 24-h temperature predictions at the target resolution. The resulting high-resolution fields and station-level forecasts are stored in SensLog and exposed via standardized APIs so that other ALIANCE [45,46,47] components can consume them.

#### 2.3.2. Infrastructure Integration

In the ALIANCE infrastructure, shown in Figure 4, SensLog acts as the core integration layer for observations, forecasts, and derived products. The workflow steps and data exchanges executed on top of this infrastructure are represented by the dataflow in Figure 4 and are detailed in Section 2.3.1.

### 2.4. Models

#### 2.4.1. Problem Formulation

The superresolution task is formulated as a supervised regression problem. For each station, a feature vector is constructed from GFS predictors, recent station observations, and static physiographic attributes. Using these inputs, the model predicts the two-meter air temperature at a lead time of twenty-four hours. The objective is to minimize the mean absolute error (MAE) between the predicted and observed station temperatures. The models are trained to predict absolute temperature values rather than temperature anomalies.

#### 2.4.2. Base Predictive Model

We evaluate several complementary model families designed for tabular geoscientific data. Specifically, we include (i) gradient-boosted decision trees (LightGBM, Microsoft, Redmond, WA, USA; https://github.com/microsoft/LightGBM, accessed on 22 December 2025) as a strong and computationally efficient tabular baseline for bias correction, (ii) a Transformer-based architecture as a modern alternative to recurrent sequence models for capturing the temporal context, implemented using PyTorch (v2.x, Meta AI, Menlo Park, CA, USA; https://pytorch.org/, accessed on 22 December 2025), (iii) TabPFN (v0.x; https://github.com/automl/TabPFN, accessed on 22 December 2025) to assess a pre-trained prior for tabular regression, and (iv) BayesNF (accessed on 22 December 2025) to represent probabilistic neural field modeling. For each base model, we additionally evaluate the physiography-conditioned KNN superresolution layer (Section 2.4.3) to obtain spatially continuous high-resolution fields. The KNN approach was chosen because it provides a simple, non-parametric way to propagate station-level predictions in a physiography-conditioned feature space, requires no assumptions about spatial stationarity, and is computationally lightweight for operational deployment.

- Bayesian Neural Fields (BayesNF)

A spatiotemporal Bayesian neural model that combines deep neural networks with hierarchical Bayesian inference. In this study, we extend its original input space (date–time and coordinates) by including static geographic predictors and selected GFS variables, while retaining MAP estimation and seasonal harmonics tuned to daily and annual cycles.

- LightGBM

A gradient-boosted decision tree model optimized for efficiency and performance on structured data. We employ LightGBM primarily as a strong non-linear tabular baseline to correct systematic GFS biases, using mostly default hyperparameters identified as robust in preliminary experiments.

- TabPFN

A Transformer-based prior-data-fitted network pre-trained on synthetic tabular tasks and adapted here for regression. TabPFN allows us to capture complex non-linear relationships between NWP predictors, static geography, and station temperatures without extensive manual feature engineering. However, TabPFN does not scale efficiently to large datasets. We therefore trained it on a random 10% subsample of the data, which may have limited its ability to exploit rare regimes. Its performance should thus be interpreted as indicative rather than fully representative of full-data training.

- Transformer for tabular bias correction

A custom Transformer architecture for structured meteorological predictors, where self-attention is applied across features rather than time. We perform a grid search over the embedding dimension, depth, number of heads, hidden layer size, and dropout to obtain an architecture that generalizes across stations.

All models are trained using identical training/validation splits and target definitions to enable the direct comparison of their skill against the GFS baseline and between model families.

#### 2.4.3. Hybrid KNN Superresolution

To transfer station-level predictions to the full high-resolution grid, we implement a hybrid KNN method:

1. For each station, a base model (LightGBM, TabPFN, Transformer, or BayesNF) is trained and used to generate a 24 h temperature forecast at the station location.
2. For each target grid cell, KNN interpolation is applied in the space of static geographical predictors (latitude, longitude, elevation, gradients). All features are normalized prior to distance computation. The optimal number of neighbors is set to the number of available stations, which empirically provided the best results, minimizing the MAE on the validation set.

This two-stage design allows the parametric models to exploit station-specific temporal information (including autoregressive inputs), while the KNN step imposes a spatial structure informed by physiography.

Conceptually, the proposed physiography-conditioned KNN propagation is related to classical spatial interpolation methods such as inverse distance weighting (IDW) and kriging. Unlike IDW, which relies solely on geometric distance, the present approach operates in a multi-dimensional feature space that includes elevation and terrain derivatives. In contrast to kriging, which assumes a parametric spatial covariance structure, the KNN layer is non-parametric and data-driven, making fewer assumptions about stationarity while remaining computationally lightweight for operational deployment.

#### 2.4.4. Training and Evaluation Protocol

To assess both temporal and spatial generalization, we use a set of spatiotemporal splits that combine the following:

- Temporal partitioning of the dataset into training and validation periods;
- Spatial partitioning into training and validation subsets of stations.

For spatial model validation, we reserve 6 randomly chosen weather stations, while the remaining 30 stations are used for training. Temporally, the training data span 2020–2022, while the validation data consist of measurements from 2023–2024.

This yields four evaluation scenarios ranging from “train–train” (training and validation on overlapping time and station subsets) to “validation–validation” (both time and stations unseen during training). In all cases, predictions are issued 24 h ahead using only information available at the forecast initialization time, which ensures that the evaluation reflects realistic operational constraints linked to GFS forecast horizons.

Model performance is quantified primarily by the mean absolute error (MAE) at station locations, with GFS values at the station grid cell used as the baseline. For the qualitative assessment of spatial realism, we also inspect predicted temperature maps over the Southern Moravia domain for selected episodes, focusing on the representation of elevation gradients, slope effects, and cold-air pooling in valleys. In this study, we report descriptive error metrics aggregated across samples and spatiotemporal splits; we therefore use terms such as “consistent” and “material” to describe effect sizes rather than formal statistical significance.

## 3. Results

### 3.1. Quantitative Evaluation on Spatiotemporal Splits

Model performance was evaluated for 24 h-ahead 2 m air temperature prediction under all combinations of temporal and spatial splits. Although some splits include “train” subsets, each configuration still represents a forecasting scenario because the target is shifted by +24 h relative to the input predictors.

The baseline GFS exhibits a mean absolute error (MAE) of between 1.66 °C and 1.88 °C across the validation configurations. All data-driven models improve upon this baseline in all split combinations, demonstrating systematic bias correction capabilities when fusing global forecast predictors with local information. TabPFN provides the lowest MAE among the standalone backbone models (typically 1.30–1.37 °C), with BayesNF, LightGBM, and Transformer exhibiting comparable but slightly higher errors depending on the split.

Table 1 reports the MAE for 24-h forecasts under all combinations of spatial and temporal splits, providing insight into each model’s generalization capabilities across both dimensions. The largest improvements over the GFS are observed for the learned models in all settings. The hybrid TabPFN-KNN configuration reaches the lowest MAE in the most challenging scenario with the validation time and validation space, achieving 1.26 °C, which corresponds to an approximately 24% reduction relative to the GFS reference. This improvement is reported as statistically significant, with paired error testing and bootstrap analysis indicating a robust performance gain.

### 3.2. Comparative Overview of Split-Dependent Performance

**Figure 5:**
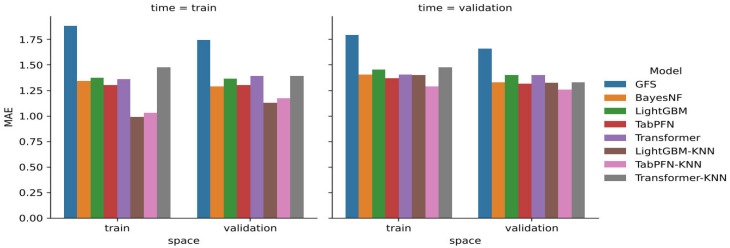
Visual comparison of the mean absolute error (MAE) of predicted temperature 24 h ahead for each possible space and time split for each model.

Figure 5 provides a compact visual comparison of the MAE across split combinations. It highlights the consistent advantage of learned backbones over the GFS, the strong performance of TabPFN among standalone models, and the benefit of adding KNN-based spatial interpolation in several split settings, particularly for TabPFN-KNN in the combined spatial and temporal validation case.

### 3.3. Time-Series Example at Validation Stations

**Figure 6:**
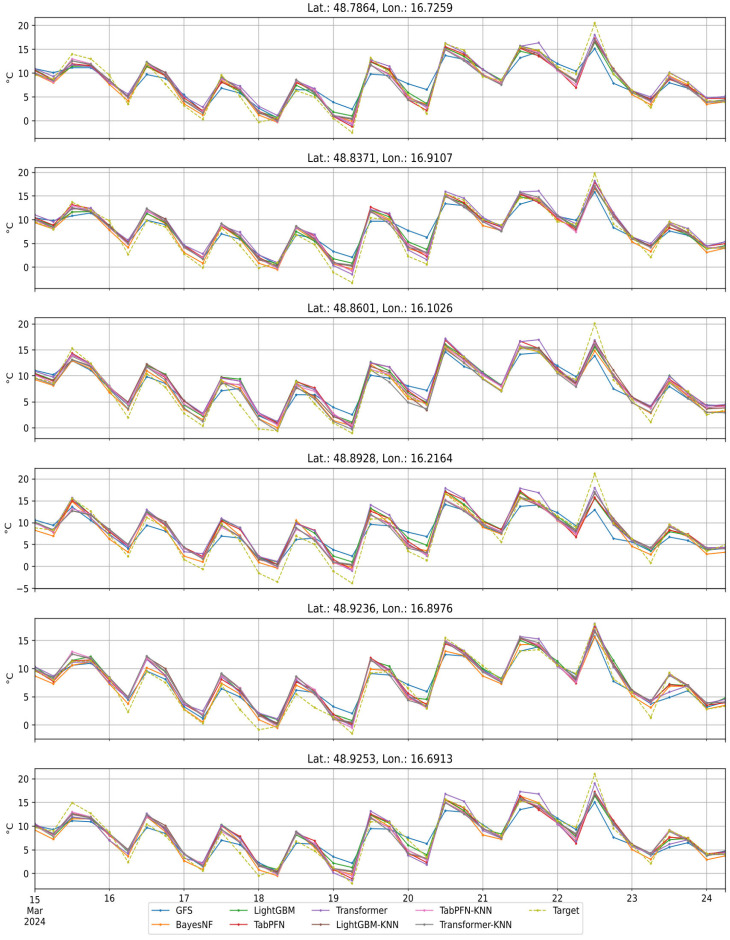
Example of predicted temperature for all models and the target temperature over a time period for each weather station in the validation split.

Figure 6 compares the predicted and observed temperature time series for representative validation stations. The plot illustrates the phase alignment of the diurnal cycle, the reduction of systematic offsets relative to the GFS, and residual errors during fast transitions such as frontal passages, where coarse predictors and sparse station coverage constrain performance.

### 3.4. Spatial Superresolution Maps (Qualitative Assessment)

**Figure 7:**
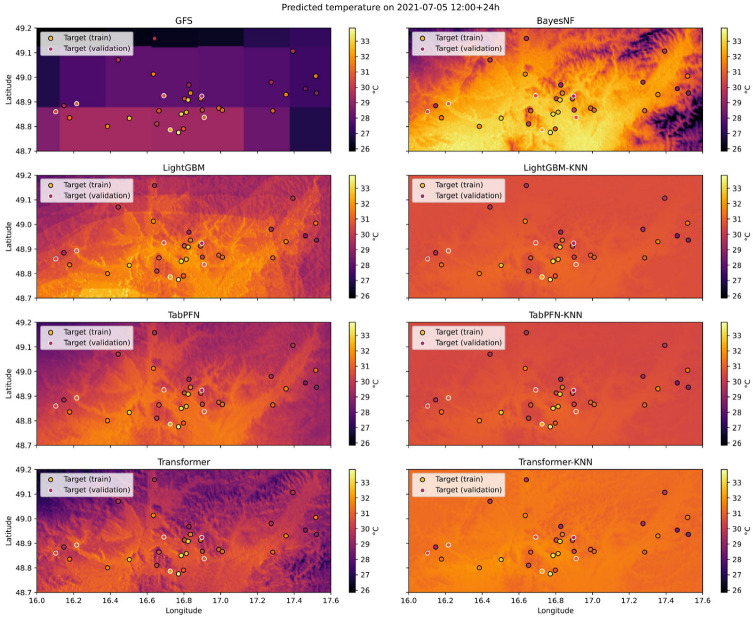
Example of predicted temperature 24 h ahead for all models from the temporal train split. Each subfigure is a different model. Target ground truth weather stations’ measurements are shown in each subfigure. Dot colors indicate temperature values according to the color scale shown in the figure. Stations from the spatial train split and validation split are highlighted with black and white outlines, respectively.

**Figure 8:**
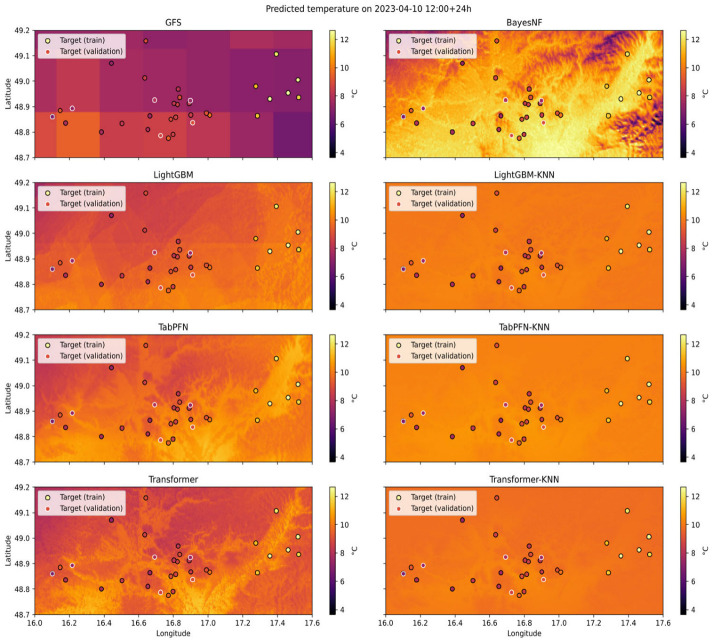
Example of predicted temperature 24 h ahead for all models from the temporal validation split. Each subfigure is a different model. Target ground truth weather stations’ measurements are shown in each subfigure. Dot colors indicate temperature values according to the color scale shown in the figure. Stations from the spatial train split and validation split are highlighted with black and white outlines, respectively.

Since a spatially continuous ground truth for the 2 m air temperature at the target resolution is not available, a qualitative assessment based on visual inspection is necessary. Figure 7 and Figure 8 are therefore used to evaluate whether the superresolved fields reproduce physically plausible and topography-driven spatial patterns. In particular, the assessment focuses on (i) monotonic temperature gradients with elevation (valley–summit contrasts), (ii) enhanced cooling in valley bottoms indicative of cold-air pooling, and (iii) asymmetries related to slope orientation and local terrain gradients.

Visual inspection indicates that all learned models reproduce the large-scale elevation-dependent temperature structure more consistently than the raw GFS baseline. Hybrid KNN-based configurations produce spatially smoother fields with coherent temperature gradients aligned with the terrain elevation. Valley regions generally exhibit lower predicted temperatures than surrounding slopes, consistent with the expected nocturnal cold-air drainage, although the strength of these minima varies across models. Slope- and aspect-related temperature contrasts are partially captured but tend to be attenuated in the KNN-propagated fields, suggesting a trade-off between spatial smoothness and the representation of localized microclimate extremes.

### 3.5. Quantitative Analysis of Spatial Gradients

To complement the qualitative assessment, we analyze the predicted temperature gradients with elevation as a simple quantitative proxy for spatial realism. For each model, temperature–altitude relationships are estimated by regressing the predicted grid cell temperatures against elevation across the domain. The resulting lapse rate-like gradients are compared between models to assess whether physically plausible cooling with height is preserved.

In addition, the spatial variance of predicted temperature fields is evaluated as an indicator of excessive smoothing. Models employing KNN-based spatial propagation exhibit lower variance than standalone backbones, confirming that the interpolation step enforces spatial continuity while also suppressing small-scale variability. This variance reduction is most pronounced in complex terrain, where localized cold-air pooling and sharp thermal contrasts are expected. These results are consistent with the visual patterns observed in Figure 7 and Figure 8 and highlight the superresolution layer’s smoothing behavior.

## 4. Discussion

### 4.1. Model Ranking and Generalization Behavior

Table 1 indicates that all learned models reduce the 24 h temperature MAE relative to the raw GFS across all spatiotemporal splits. Considering the most operationally relevant regime—unseen stations and an unseen future period (time = validation, space = validation)—the best-performing configuration is TabPFN-KNN (MAE 1.26 °C). This corresponds to an error reduction of 0.40 °C (≈24%) compared with the GFS baseline (1.66 °C). The next-best group is formed by TabPFN (1.32 °C), LightGBM-KNN (1.32 °C), and BayesNF (1.33 °C), all providing approximately 20% improvements over the GFS, followed by LightGBM and Transformer (both 1.40 °C).

In regimes with known stations (space = train), the differences between backbones are comparatively small, with TabPFN and BayesNF remaining consistently among the best. In the easiest split (time = train, space = train), LightGBM-KNN reaches the lowest MAE (0.99 °C); however, this configuration benefits from maximal station representativeness and does not quantify generalization to new stations or unseen seasons. For model selection aimed at operational deployment, results obtained in the validation/validation setting should therefore be treated as primary.

### 4.2. Interpretation of the KNN Superresolution Effect

The KNN stage propagates station-level forecasts onto a high-resolution grid using similarity in a static physiographic space. Its impact is split-dependent. It yields the largest gains when the evaluation includes locations outside the training station subset but within the training time window (time = train, space = validation), indicating that spatial propagation contributes substantially when temporal variability is already well represented. In the most demanding regime (time = validation, space = validation), the additional improvement of TabPFN-KNN over TabPFN demonstrates that the spatial propagation also adds value under simultaneous temporal and spatial generalization.

To further quantify the incremental contribution of the physiography-conditioned KNN propagation, we compare each backbone to its KNN-augmented variant (Table 2). Because the propagation operates in a static physiographic feature space (elevation and terrain gradients), this comparison isolates the impact of physiographic descriptors on spatial generalization. The largest MAE reductions occur when stations are withheld (space = validation), e.g., TabPFN improves by 0.13 °C (time = train, space = validation) and by 0.06 °C (time = validation, space = validation), consistent with physiography-informed similarity mitigating location-dependent biases (Table 3).

At the same time, KNN interpolation tends to smooth spatial fields. While this can reduce the average station error, it may attenuate localized microclimate extremes (e.g., cold-air pooling). Consequently, KNN should be considered an operational superresolution layer rather than a physically explicit spatial model. Spatial plausibility therefore requires complementary qualitative checks and, where possible, spatially distributed reference observations.

### 4.3. Practical Recommendations for Operational Deployment

Based on Table 1, TabPFN-KNN is the preferred default configuration for the operational production of high-resolution temperature fields, because it yields the lowest MAE in the validation/validation regime while providing spatially continuous outputs. If only station-level forecasts are required, the standalone TabPFN model provides stable accuracy across split configurations and can be deployed with lower system complexity. BayesNF remains competitive and may be preferred when stronger terrain dependence in spatial patterns is required, noting that its potential advantage may be underestimated by the station-only MAE.

Future evaluation should incorporate spatial reference information (e.g., independent stations, mobile transects, or satellite thermal observations with an explicit air temperature conversion model) to quantify the trade-off between smoothness and microclimate detail and to validate the superresolved fields beyond point locations.

### 4.4. Comparison with Related Work and Novelty

From a precision agriculture perspective, systematic reviews of AI-enabled yield prediction and sustainable farming identify weather and climate information as among the most influential inputs, yet meteorological forcing is often derived from coarse NWP/reanalysis products or sparse station data, limiting field-scale decision support [48,49]. Representative yield prediction studies explicitly integrate temperature and precipitation covariates but focus on agronomic response models rather than on improving the meteorological forcing itself [50,51,52]. Recent work on hyperlocal, IoT-supported weather forecasting further emphasizes the need for high-resolution, near-real-time meteorological fields for smart farming applications [44]. The present study addresses this gap by providing an operationally deployable NWP post-processing and superresolution workflow that conditions spatial propagation on physiographic similarity, producing spatially continuous, high-resolution temperature maps intended as inputs to downstream agronomic models and decision support pipelines.

Recent work on the machine learning-based post-processing of numerical weather prediction has shown that learning situation-dependent biases can improve forecast skill across variables and lead times, with reported gains typically in the 10–15% range in the RMSE for global medium-range systems. While direct comparison is limited by differences in region, variables, lead time, and error metrics, the approximately 24% MAE reduction observed here for the 24 h 2 min air temperature (relative to the raw GFS in the time = validation, space = validation regime) is consistent with the magnitude of improvements reported in recent operational post-processing studies and surveys [21,22,23,24].

### 4.5. Evaluation of Hypotheses

H1 is supported by the quantitative evaluation, because all learned configurations reduce the station-level MAE relative to the raw GFS across the spatiotemporal splits reported in Table 1, with the corresponding RMSE results given in Table 2.

H2 is supported by the comparison between backbones and their KNN-augmented variants in Table 1, particularly in regimes involving spatial generalization. In the validation time and validation space regime, TabPFN-KNN improves the MAE from 1.32 °C to 1.26 °C, and LightGBM-KNN improves the MAE from 1.40 °C to 1.32 °C, which is consistent with physiography-informed propagation reducing location-dependent biases.

H3 is partially supported by the stable performance observed under temporal and spatial holdout splits, including the validation time and validation space regime. However, this work does not yet provide dedicated experiments considering station availability constraints such as controlled missingness or heterogeneous sampling, and this aspect remains a priority for future validation.

### 4.6. Strengths and Limitations

The main strength of the proposed approach is its operational orientation: it combines routinely available GFS predictors with regional station observations and static physiographic descriptors to produce spatially continuous, high-resolution 24 h 2 m air temperature fields suitable for precision agriculture. Across all spatiotemporal split configurations, the learned models consistently reduce the error relative to the raw GFS; in the most operationally relevant regime (time = validation, space = validation), TabPFN-KNN achieves MAE = 1.26 °C, compared to 1.66 °C for GFS (about 24% reduction). Key limitations are that the evaluation is necessarily station-based, i.e., a spatially continuous reference air temperature at the target grid resolution is not available, and that the present experiments are restricted to one region and a 24 h horizon. Finally, the KNN propagation enforces smooth spatial fields and may attenuate localized extremes (e.g., cold-air pooling), motivating complementary spatial validation and/or more explicit spatial modeling in future work.

## 5. Conclusions

This work addresses the problem of producing operationally useful, field-scale near-surface air temperature forecasts by fusing coarse global NWP predictors with local observations and a static physiographic context and by transforming station-level forecasts into spatially continuous high-resolution fields. The core contribution is an end-to-end workflow that couples machine learning-based 24 h bias correction at station locations with a geographically conditioned superresolution step, designed for integration into sensor data infrastructures.

Quantitative results demonstrate that all learned models outperform the raw GFS baseline across all spatiotemporal evaluation splits. In the most operationally relevant regime—simultaneous generalization to unseen stations and unseen future periods (time = validation, space = validation)—the best configuration was TabPFN-KNN (MAE 1.26 °C), corresponding to an approximately 24% reduction in the MAE compared with GFS (1.66 °C). Standalone TabPFN, LightGBM-KNN, and BayesNF formed a competitive second tier, while LightGBM and Transformer provided consistent but smaller improvements. These findings support the feasibility of superresolved temperature products driven by routinely available global forecasts and regional station networks.

From an operational perspective, TabPFN-KNN is recommended as the default configuration when spatially continuous temperature fields are required. If the application requires only station-level forecasts, a standalone TabPFN configuration provides robust performance with reduced system complexity. The KNN-based spatial propagation improves map continuity and, in several configurations, further reduces station errors; however, its smoothing behavior motivates complementary spatial validation when microclimate extremes are critical for decision support.

Limitations are primarily linked to validation constraints. The point station MAE does not fully characterize the spatial realism of superresolved fields, particularly in complex terrain and under stable boundary layer conditions. Future work should therefore incorporate spatial reference information (e.g., independent stations, mobile transects, or satellite thermal observations combined with a dedicated air temperature conversion model), extend the approach to additional variables relevant for agriculture (humidity, precipitation, wind), and evaluate its robustness under realistic station availability and missingness. A structured evaluation of hypotheses H1–H3 is provided in Section 4.5.

## 6. Data, Software, and Reproducibility

All primary input datasets used in this study are publicly available. Global Forecast System predictors were obtained from the NCAR Research Data Archive, station observations from the agricultural weather station network are accessible through the SensLog platform, and the static physiographic layers were derived from the ASTER Global Digital Elevation Model. The modeling workflow, feature construction, and evaluation protocol are described in sufficient detail in Section 2, Section 3 and Section 4 to enable independent reimplementation and comparative analyses. The source code and the operational data-processing pipeline are not released publicly because the implementation is currently integrated into commercial applications.

Reproducibility is addressed at the level of inputs, methodological specification, and evaluation. Because the external datasets can be obtained from the cited repositories, and because the predictor set, temporal coverage, spatial domain, and train validation splits are defined in the manuscript, other researchers can reconstruct an equivalent experimental dataset and repeat the modeling and assessment steps. An independent implementation of the described feature engineering, learning algorithms, and superresolution mapping should therefore yield comparable performance against the same baseline, although small numerical differences can occur for models trained with stochastic optimization. Exact computational reproducibility, i.e., the execution of the original software pipeline to regenerate identical intermediate products and model artifacts, is currently not possible because the production implementation and its configuration are embedded in commercial applications and are not distributed.

## Figures and Tables

**Table 1:** Mean absolute error (MAE) of predicted temperature 24 h ahead for each possible space and time split for each model.

Model	Time = Train, Space = Train	Time = Train, Space = Validation	Time = Validation, Space = Train	Time = Validation, Space = Validation
GFS	1.88	1.75	1.79	1.66
BayesNF	1.34	1.29	1.40	1.33
LightGBM	1.37	1.37	1.46	1.40
TabPFN	1.30	1.30	1.37	1.32
Transformer	1.36	1.39	1.40	1.40
LightGBM-KNN	0.99	1.13	1.40	1.32
TabPFN-KNN	1.03	1.17	1.29	1.26
Transformer-KNN	1.48	1.39	1.48	1.33

**Table 2:** Root mean squared error (RMSE) of predicted temperature 24 h ahead for each possible space and time split for each model.

Model	Time = Train, Space = Train	Time = Train, Space = Validation	Time = Validation, Space = Train	Time = Validation, Space = Validation
GFS	2.48	2.40	2.40	2.24
BayesNF	1.82	1.88	1.86	1.77
LightGBM	1.82	1.92	1.96	1.90
TabPFN	1.78	1.87	1.87	1.80
Transformer	1.86	1.97	1.90	1.90
LightGBM-KNN	1.30	1.65	1.87	1.80
TabPFN-KNN	1.41	1.72	1.75	1.73
Transformer-KNN	1.92	1.99	1.94	1.83

**Table 3:** MAE reduction due to physiography-conditioned KNN propagation (ΔMAE = MAE_base − MAE_base-KNN; positive values indicate improvement).

Backbone	Time = Train, Space = Train	Time = Train, Space = Validation	Time = Validation, Space = Train	Time = Validation, Space = Validation
LightGBM	0.38	0.24	0.06	0.08
TabPFN	0.27	0.13	0.08	0.06
Transformer	−0.12	0.00	−0.08	0.07

## Data Availability

Publicly available datasets were analyzed in this study. Global Forecast System (GFS) forecast data are available from the NCAR Research Data Archive (https://rda.ucar.edu/ accessed on 22 December 2025). ERA5-Land reanalysis data are available from the Copernicus Climate Data Store (https://cds.climate.copernicus.eu/ accessed on 22 December 2025). Station observations from the agricultural weather station network operated by the National Wine Growers’ Association (EKOVÍN) are accessible via the SensLog platform and are available from the data providers upon reasonable request, subject to data provider restrictions. Static physiographic data were derived from the ASTER Global Digital Elevation Model (NASA/METI), available at https://lpdaac.usgs.gov/products/astgtmv003/ accessed on 22 December 2025.

## Abbreviations

The following abbreviations are used in this manuscript:

ALIANCE	Advanced Lightweight Infrastructure for Agriculture through Novel Computing and Environmental Services
ASTGTM	ASTER Global Digital Elevation Model
CTU	Czech Technical University in Prague
ERA5	Fifth-Generation ECMWF Atmospheric Reanalysis
ERA5-Land	ERA5 Dataset Focused on Land Variables
EO	Earth Observation
GFS	Global Forecast System (GFS; National Centers for Environmental Prediction, National Oceanic and Atmospheric Administration—NOAA, College Park, MD, USA)
KNN	K-Nearest Neighbors
MAE	Mean Absolute Error
ML	Machine Learning
NWP	Numerical Weather Prediction
SensLog	Web-based sensor data management system (International consortium (Czech Republic, Latvia))
SHAP	SHapley Additive exPlanations
TabPFN	Tabular Prior-Data-Fitted Network
TA ČR	Technology Agency of the Czech Republic
U-Net	Convolutional Neural Network Architecture for Image Segmentation
